# Effect of alfalfa substituted with ramie on the expression of apoptotic genes in the gastrointestinal tracts of goats

**DOI:** 10.1002/fsn3.2848

**Published:** 2022-04-01

**Authors:** Qian Liu, Chao Fu, Hai Yang, Chuanshe Zhou, Jinhe Kang, Liang Chen, Zhiwei Kong, Zhiliang Tan, Shaoxun Tang

**Affiliations:** ^1^ College of Life Science and Environment Hengyang Normal University Hengyang China; ^2^ Key Laboratory for Agro‐Ecological Processes in Subtropical Region National Engineering Laboratory for Pollution Control and Waste Utilization in Livestock and Poultry Production Hunan Provincial Key Laboratory of Animal Nutritional Physiology and Metabolic Process Institute of Subtropical Agriculture Chinese Academy of Sciences Changsha China; ^3^ Jiangxi Academy of Forestry Nanchang China; ^4^ University of Chinese Academy of Sciences Beijing China

**Keywords:** alfalfa, apoptosis, gastrointestinal tract, goats, ramie

## Abstract

The study investigated the effect of alfalfa hay substituted with ramie silage on the expression of apoptotic genes in the gastrointestinal tract of goats. Thirty‐two goats were randomly allocated into four groups, in which the alfalfa was substituted with ramie at 0%, 35%, 75%, and 100% levels, respectively. In the rumen, the mRNA expression of Bax was significantly up‐regulated (*p* = .0007) when alfalfa was 100% substituted by ramie; the mRNA expression of Bcl‐2/Bax was significantly down‐regulated (*p* = .02) when alfalfa was 100% substituted by ramie compared with the 75% substituted treatment; the protein expression of Bcl‐xl was significantly down‐regulated (*p* = .03) when alfalfa was 100% substituted by ramie compared with 35% and 75% substituted treatments, respectively. In the jejunum, the mRNA expression of p53 was significantly up‐regulated (*p* = .01) when alfalfa was 100% substituted by ramie compared with 0% and 35% substituted treatments; the protein expression of p53 was significantly up‐regulated (*p* = .001) when alfalfa was 35% substituted by ramie compared with 0% and 75% substituted treatments. However, the activity of Caspase‐3 was not affected by different substituting levels of ramie in the rumen and jejunum of goats (*p* > .05). In conclusion, ramie with high substitution had strong antinutritional effect, which might promote the apoptosis in the gastrointestinal tract of goats in a caspase‐independent manner, thus affecting the growth and development of goat. It was suggested that ramie should not replace alfalfa more than 35% in the process of goat feeding.

## Graphical abstract

This study aimed to explore the effect of ramie on the expression of apoptosis genes in goats, the results indicated that ramie silage should not replace alfalfa more than 35% in the process of goat feeding. Provided theory and data support for new feed resource exploitation for ruminant husbandry industry.

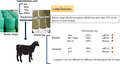

## 1 INTRODUCTION

Apoptosis, known as programmed cell death, is common and essential at all stages of life in animals. Apoptosis is closely related to the growth and development of living organisms (Zhan & Tang, 2014), the removal of damaged and senile cells (Bailey et al., 2002), and the prevention of cancer cells (Hsu et al., 2004). It is a basic biological phenomenon of cells (Fadeel & Orrenius, 2005). There are two kinds of genes affecting cell apoptosis, namely, pro‐apoptotic genes and antiapoptotic genes (Mischiati et al., 2003). The pro‐apoptotic genes, such as Bax, Bad, Bid, Bcl‐xs, and p53, can trigger or promote apoptosis, whereas the antiapoptotic members, including Bcl‐2, Bcl‐xl, and Bcl‐w, can protect the cell by preventing normal cell death (Reshi et al., 2017; Youle, 2008). Bcl‐2 is a proto‐oncogene with a pivotal role in apoptosis, which prevents apoptosis by inhibiting the release of cytochrome C from mitochondria (Wang et al., 2015), while Bax is the main mediator of the mitochondrial pathway by promoting the release of cytochrome C from the mitochondria (Eskes et al., 2000). Bax and Bcl‐2 can form dimer, when Bax dimer is dominant, apoptosis is induced; when Bcl‐2 dimer is dominant, apoptosis is inhibited (Verma et al., 2014). Bcl‐xl exerts an antiapoptotic effect by blocking the destruction of the outer mitochondrial membrane by Bax (Liu et al., 2016), and p53 is essentially a nuclear phosphate protein that regulates the expression of related genes in the form of transcription factor, promotes cell cycle arrest, and induces apoptosis (Guicciardi et al., 2000).

Apoptosis is one of the important research directions of animal nutrition and induced by many factors, such as high‐temperature stimulation, hormone and growth factor imbalance, antinutritional factor stimulation, bacterial and viral infection, and so on (Brockmann et al., 2014; Zarzynska et al., 2005). The apoptosis of the gastrointestinal tract (GIT) was closely related to the regeneration and repair of gastrointestinal cells in animals (Zhu et al., 2011). Some diets contain antinutritional factors, so the diet is an important factor affecting apoptosis in GIT. At present, alfalfa is one of the important protein sources for ruminants. It is known as the "King of herbage" because of its high yield, high protein content, strong adaptability, high soil improvement, and economic value (Iannucci et al., 2002). However, it is not suitable for growth in southern China, and its planting range is limited. Ramie has a strong ability of environmental stress resistance, and its yield is much higher than that of alfalfa in the southern region of China (Zhu et al., 2016). In addition, ramie is rich in crude protein, crude fat, crude fiber, carotenoids, vitamins, and animal growth‐related elements, of which crude protein, lysine, and calcium are generally higher than those of alfalfa (Squibb et al., 2010; Wu et al., 2017). Gao et al. (2016) showed that ramie could improve meat quality and growth performance of Boer crossbreed goats. Therefore, ramie has great potential as a new feed resource for ruminants. In addition, in vitro experiment showed that the addition of silage ramie leaves and stem of ramie leaf would increase the nutrient digestibility, and dietary supplemented 40% of silage ramie leaves, the nutrient digestibility was the highest, the content of VFA in leaves of ramie silage also higher than other groups (Despal, 2010). Generally speaking, ramie has strong advantages in nutrition, digestibility, and economic benefits as a forage.

Previous studies showed that ramie silage could replace alfalfa hay in the diet of dairy cow without affecting production performance, milk composition, and serum index, and the suitable replacement ratio was 33%–67% in the ration of dairy cows (Dai et al., 2018; Wu et al., 2017). Meanwhile, our previous study (Tang et al., 2019) showed that the nutrient digestion of CP, NDF, ADF, Ca, and ash and the body weight gain of goats would decline as the proportion of ramie substitution for alfalfa exceeds 70%. The digestibility of CP was positively correlated with the length of intestinal villi (Liu et al., 2009; Salgado et al., 2002), and typical morphological characteristics resulted from apoptosis include shortening or even disappearance of intestinal villi (Jones & Gores, 1998); however, the molecular mechanism of ramie replacing alfalfa was rarely studied so far. Therefore, we hypothesized that the substitution of ramie for alfalfa could affect the apoptosis of GIT of goats. The objective of this study was to explore the effect of ramie on the expression of apoptosis genes in GIT of goats, and to provide theory and data support for new feed resource exploitation for ruminant husbandry industry.

## 2 MATERIALS AND METHODS

The experiments were conducted according to the animal care guidelines of the Animal Care Committee, Institute of Subtropical Agriculture, Chinese Academy of Sciences, Changsha City, Hunan Province, China (No. KYNEAAM‐2006‐0015).

### 2.1 Animal preparation and experimental design

The experiment was conducted with 32 individually housed *Xiangdong* black goats (a local goat breed in southern China, age: 1.2 ± 0.2 years; body weight: 16.0 ± 2.0 kg). To ensure that the environmental conditions were similar throughout the experiment, all the goats were housed in a well‐ventilated room with controlled temperature (25 ± 3°C) and humidity (60% ± 2%). In the present study, 32 goats were randomly separated into four groups, the proportion of ramie in substitutes for alfalfa was 0% (A), 35% (B), 75% (C), and 100% (D). The composition of experimental diets is shown in Table 1. The nutrient intake, fecal excretion, and nutrient digestibility of goats were reported in our companion work, including DM (dry matter), GE (gross energy), NDF (neutral detergent fiber), CP (crude protein), ADF (acid detergent fiber), P (phosphorous), and Ash (Tang et al., 2019).

**TABLE 1:** Ingredients and chemical composition of diet

Ingredient (g/kg DM)	Treat^a^				Alfalfa	Ramie
	A	B	C	D		
Corn	100	180	245	280		
Wheat bran	255	180	110	70		
Fatty powder	15	8	10	15		
Calcium hydrophosphate	5	7	10	10		
Salt	5	5	5	5		
Mineral and vitamin premix^b^	20	20	20	20		
Rice straw	240	240	240	240		
Alfalfa	360	234	108	0		
Ramie	0	126	252	360		
Chemical composition (g/kg DM)						
ME^c^ (MJ/Kg)	8.54	8.58	8.62	8.75	/	/
CP	115	116	111	111	137	173
NDF	479	488	478	459	500	621
ADF	286	275	265	261	411	449
Ca	27.3	26	28.5	28.9	31.2	36.2
P	1.74	1.84	2.22	2.35	4.33	2.75
Ash	94.3	97.2	107	111	83.6	138

^a^ A, B, C, and D mean the percentage of alfalfa substituted with ramie was 0%, 35%, 75%, and 100%, respectively.

^b^ Premix per kg: 5.88 g FeSO_4_ 7H_2_O, 2.33 g CuSO_4_ 5H_2_O, 11.96 g MnSO_4_ H_2_O, 8.29 g ZnSO_4_ H_2_O, 20 mg Na_2_SeO_3_, 50 mg KI, 35 mg CoCl_2_ 6H_2_O, 90,000 IU vitamin A, 17,000 IU vitamin D, and 17,500 IU vitamin.

^c^ ME was calculated according to Mehtiö et al. (2018).

### 2.2 Sample collection

After 6 weeks of feeding, all goats were euthanized by captive bolt stunning and exsanguinated. Immediately, the abdominal cavity was opened and the GIT was removed. The GIT was rinsed with precooled saline, and then tissue samples of bottom of the rumen and the middle jejunum were collected, wrapped with sterilized tinfoil, and frozen in liquid nitrogen, and stored at −80°C until later use. The rumen and jejunum, which differed in structure and function, were selected to represent the forestomach and intestine of ruminants.

### 2.3 RNA isolation, cDNA preparation and quantitative real‐time PCR analysis

Total RNA (*n* = 8 for each group) was extracted using TRIZOL (Invitrogen) according to the manufacturer's instructions. After genomic DNA was eliminated by digestion with DNase I (Thermo Scientific), the RNA quality and quantity were determined using NanoDrop 2000 (Thermo Scientific). All RNA samples showed A260/A280 values within the range of 2.01–2.06 and A260/A230 values above 2.0. The integrity of collected RNA was analyzed with gel electrophoresis. Afterwards, 1 μg of the extracted RNA was immediately reverse‐transcribed to synthesize tissue‐specific cDNA using PrimeScript™ RT reagent Kit (Takara). The reverse transcription procedures were conducted according to the modified steps by Ran et al. (2016). The prepared cDNA samples were further purified, quantified, and diluted to the same initial concentration, and stored at −20°C until subsequent quantitative real‐time PCR analysis.

Primers were designed using the Primer‐Primer 5 and online tools Primer 3 (http://simgene.com/Primer3) and are listed in Table 2. β‐Actin were selected as reference genes to normalize the expression of target genes. The specificity of designed primers was checked via online Primer‐BLAST (NCBI) and subsequent gel electrophoresis analyzing and sequencing of PCR products of designed primers, as well as melt curve analyzed during quantitative real‐time PCR. The quantitative real‐time PCR was performed on an ABI‐7900HT qPCR system (Applied Biosystems) using FG POWER SYBR GREEN PCR MASTER MIX (Applied Biosystems). Quantification of the PCR products of all target genes was evaluated in comparison with the PCR products of β‐Actin and the amplification efficiency of target gene and reference gene is close to 100% and the efficiency deviation between them is within 5%. The relative changes in mRNA expression levels were calculated according to the 2 ^−△△CT^ method (Livak & Schmittgen, 2001), where −ΔΔ CT = −(Δ CT _samples at other treatments_ − Δ CT _samples at control group_) and Δ CT = (CT _samples_ − CT _β–actin_)/2. The control group in the rumen was used for the standardization.

**TABLE 2:** Primers for quantitative real‐time PCR (qRT‐PCR)

Primer	Sense	Sequence	Product size
β‐actin	Forward	5′‐ATGGCTACTGCTGCGTCGT‐3′	263 bp
	Reverse	5′‐TTGAAGGTGGTCTCGTGGAT‐3′	
Bcl‐2	Forward	5′‐ATGACCGAGTACCTGAACC‐3′	148 bp
	Reverse	5′‐AGACAGCCAGGAGAAATCA‐3′	
Bax	Forward	5′‐CGACGGCAACTTCAA‐3′	165 bp
	Reverse	5′‐CACTCCAGCCACAAAG‐3′	
Bcl‐xl	Forward	5′‐CCCGCTCTTCATCTT‐3′	157 bp
	Reverse	5′‐CTCTGGGCGTGTATCT‐3′	
p53	Forward	5′‐CTGTCGTCCTTTGTCC‐3′	132 bp
	Reverse	5′‐TGCTCCAGCTTCTTGTA‐3′	

### 2.4 Caspase‐3 activity assay

Activity of Caspase‐3 was measured using Caspase‐3 activity assay kit (Beyotime, C1116) according to the manufacturer's instructions. In brief, samples were ground with liquid nitrogen, resuspended in lysis buffer, and left on ice for 5 min. Firstly, the lysate was centrifuged at 18,000 *g* for 15 min at 4°C, the supernatant was taken in an ice bath. Secondly, 10 μl Ac‐DEVDpNA (2 mM) was added and mixed, and incubated at 37°C for 1 h. Finally, Caspase‐3 activity was measured. The release of p‐nitroanilide (pNA) was measured with Multiskan Spectrum (Thermo Scientific) at an absorbance of 405 nm. One unit is the amount of enzyme that will cleave 1.0 nmol of the colorimetric substrate Ac‐DEVD‐pNA per hour at 37℃ under saturated substrate concentrations.

### 2.5 Western blot analysis

Protein isolation and western blot analysis were performed as described previously (Karaki et al., 2006; Yan et al., 2015). Specifically, mucosal samples (approximately 0.2 g each) were frozen in liquid nitrogen, pulverized to a powder using a mortar and pestle, dissolved in 300 μl of RIPA lysis solution (mixed with 1% protease inhibitor), and then lysed on ice for 30 min. The sample was then centrifuged at 12,000 *g* for 15 min at 4°C and the supernatant was obtained as the desired protein sample. Protein quantification was carried out using the BCA method to determine protein concentration. The sample volume was then calculated and mixed with 5 × loading buffer, incubated at 95°C for 5 min to denature the protein, and stored at −20°C.

The same amount of protein was separated from each sample and prestained standard (Bio‐Rad Laboratories) by electrophoresis in a 10% SDS‐polyacrylamide gel. The isolated proteins were transferred onto polyvinylidene fluoride (PVDF) membranes (Bio‐Rad Laboratories) at a constant current of 200 mA for 70 min. The PVDF membranes were then incubated for 1 h with 5% skim milk in TBS buffer containing 0.2% Tween 20. The pre‐blocked membranes were diluted 1:200 in 1 × TBST overnight at 4°C with anti‐bcl‐2 antibody (12789–1‐AP, Proteintech, Rabbit polyclonal, 1:3000), anti‐bcl‐xl antibody (10783–1‐AP, Proteintech, Rabbit polyclonal, 1:3000), anti‐bax antibody (50599–2‐Ig, Proteintech, Rabbit polyclonal,1:3000), anti‐p53 antibody (10442–1‐AP, Proteintech, Rabbit polyclonal,1:3000), and anti‐β‐actin antibody (60008–1‐Ig, Proteintech, Mouse monoclonal, 1:5000), washed three times in 1 × TBST for 15 min each. Incubated with horseradish peroxidase (HRP)‐labeled secondary antibody (1:3000; Proteintech Group, Inc.) in 1 × TBST for 1 h at room temperature, and then washed three times in 1 × TBST for 10 min each time. Bcl‐2 and Bax, Bcl‐xl, and p53 protein bands were detected with Western Bright ECL Western Blotting HRP substrate (APGBio) and photographed with an AlphaImager 2200 digital imaging system.

### 2.6 Statistical analyses

Statistical analyses of data were evaluated through a one‐way ANOVA procedure, and animals were used as experimental unit. Statistical significance was set at *p* < .05 and tendencies at .05 ≤ *p* ≤ .10. All statistical analyses were conducted with JMPR 12.1.0. (SAS Institute Inc.).

## 3 RESULTS

### 3.1 Caspase‐3 activity in different parts of GIT of goats

The activities of Caspase‐3 in the rumen and jejunum were not affected (*p* > .05) by different substituting levels of ramie of goats (Table 3). However, the activity of Caspase‐3 was numerically increased in the rumen while it was numerically decreased in the jejunum for the D groups compared to A group (Table 3).

**TABLE 3:** Effects of different substitution levels of ramie to alfalfa on the activity of Caspase‐3 enzyme (U) in different parts of GIT of goats (*n* = 8)

Tract	Treat^a^				SEM	*p*
	A	B	C	D		
Rumen	10.96	16.41	14.56	14.98	2.189	.362
Jejunum	36.90	31.99	33.33	32.80	2.152	.936

Abbreviation: SEM, Standard error of mean.

^a^ A, B, C, and D mean the percentage of alfalfa substituted with ramie was 0%, 35%, 75%, and 100%, respectively.

### 3.2 mRNA expression of apoptosis‐related genes

In the rumen, Bcl‐xl and Bax expression in group D was significantly up‐regulated compared to group A (*p* = .004) (Table 4); while Bcl‐xl and Bax did not affect in groups B and C compared with group A; Bcl‐2/Bax was significantly down‐regulated (*p* = .02) when alfalfa in group D compared with the group C. In the jejunum, p53 was significantly up‐regulated (*p* = .01) in group D compared with the group A, while it did not affect in groups B and C compared with group A. Bax, Bcl‐xl, Bcl‐2, and the ratio of Bcl‐2/Bax in the jejunum were not significantly different among four groups (*p* > .05).

**TABLE 4:** Effects of different substitution levels of ramie to alfalfa on the mRNA expression of apoptosis‐related genes in GIT of goats (*n* = 8)

Items	Treat^1^				SEM	*p*
	A	B	C	D		
Rumen						
Bcl‐2	1.09	1.46	1.25	0.98	0.212	.460
Bcl‐xl	1.07^b^	2.06^ab^	1.61^b^	3.32^a^	0.376	.004
p53	1.12	1.85	1.26	1.46	0.324	.440
Bax	1.26^b^	1.06^b^	0.65^b^	3.35^a^	0.373	.001
Bcl‐2/Bax	1.31^ab^	1.57^ab^	3.30^a^	0.32^b^	0.569	.021
Jejunum						
Bcl‐2	1.06	0.66	2.01	0.66	0.376	.070
Bcl‐xl	1.20	1.31	1.69	0.27	0.433	.172
p53	1.38^b^	1.74^b^	1.76^ab^	3.47^a^	0.397	.012
Bax	1.11	0.48	2.23	3.14	0.778	.293
Bcl‐2/Bax	1.25	1.52	0.43	0.45	0.293	.060

Note

^a,b^ Means in the same row without a common superscript differ (*p* < .05).

Abbreviation: SEM = Standard error of mean.

^1^ A, B, C, and D mean the percentage of alfalfa substituted with ramie was 0%, 35%, 75%, and 100%, respectively.

### 3.3 Protein expression of apoptosis‐related genes

**FIGURE 1:**
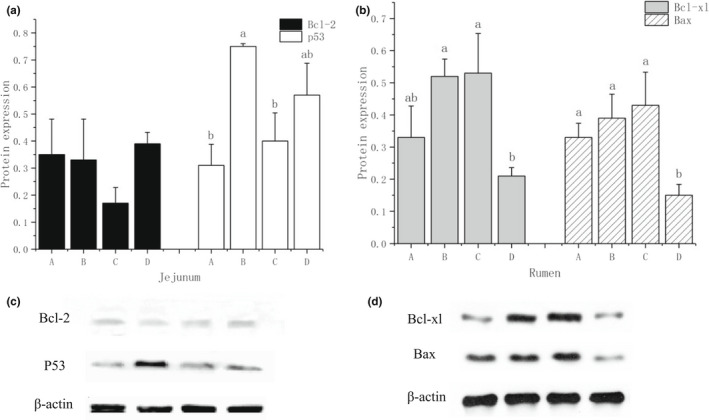
(a) Effect of ramie treatments on the protein expression of apoptosis‐related proteins in jejunum of goats; (b) Effect of ramie treatments on the protein expression of apoptosis‐related proteins in rumen of goats; (c) Imprint of apoptosis‐related proteins in the jejunum of goats under different ramie treatments; (d) Imprint of apoptosis‐related proteins in the rumen of goats under different ramie treatments

In the rumen, Bcl‐xl was significantly down‐regulated (*p* = .03) in group D compared with groups B and C, respectively; Bax was significantly down‐regulated (*p* = .002) in group D comparing with other three groups (Figure 1b,d). In the jejunum, p53 was significantly up‐regulated (*p* = .001) in group B compared with groups A and C. There was no significant difference in the protein expression level of Bcl‐2 among four groups in the jejunum (*p* > .05) (Figure 1a,c).

## 4 DISCUSSION

Previous studies have shown that the intestinal villi became shorter or even disappear in the process of apoptosis (Jones & Gores, 1998), and the indexes such as crude protein digestibility were positively related to the length of intestinal villi (Liu et al., 2009; Salgado et al., 2002). Therefore, the indexes such as crude protein digestibility are closely related to the occurrence of apoptosis. Our previous studies showed that the growth index, digestibility of CP, NDF, ADF, Ash, and Ca of D group were significantly lower than those of the other three groups (Tang et al., 2019). In terms of rumen ecology, although the volatile fatty acid profile was not affected, the ammonia concentration was declined linearly by ramie substitution (Tang et al., 2019). Therefore, on the physiological level, we speculated that ramie substitution could affect the apoptosis of gastrointestinal cells to a certain extent. In this study, we found that mRNA expression of Bcl‐2/Bax and protein expression of Bcl‐xl decreased in the rumen of group D, while mRNA expression of p53 increased in the jejunum. This suggests that 100% ramie substitution may promote apoptosis of gastrointestinal cells. Overall, the pro‐apoptotic effect was more pronounced in the high substitution rate ramie group than in the low substitution rate ramie group. The reason might be that ramie contains excessive crude fiber, mannose, pectin, Ca, and other substances, which made ramie had certain antinutritional effect (Ray et al., 2014). High ramie substitution rate was difficult to be effectively digested in GIT of goats, and the high content of Ca in ramie might affect the balance of Ca and P in animal diets (Liu, 2018), while the antinutritional effect of ramie could affect the digestive rate of GIT of goats. If the nutritional digestibility was too low, the body would suffer from malnutrition, and it would affect the biosynthesis of oxidase and the level of endogenous antioxidant, so as to increase the production of free radicals, the DNA content of intestinal cells, the protein synthesis, and cell proliferation, while could activate the autophagy pathway and promote apoptosis (Zhu et al., 2011), thus affected the expression of Bcl‐2/Bax, Bcl‐xl, p53, and other apoptosis genes. Strangely, we found that the expression of p53 protein in 35% ramie substitution group was significantly higher than that in 0% and 75% ramie substitution group. This result was not completely consistent with our inference. The reason might be that when the cells were stimulated by mild physiological stress, p53 would play a role in DNA repair. When the cells were stimulated by severe stress such as the activated proto oncogene, p53 protein would start the apoptosis process and cause apoptosis (Kang et al., 2009; Wang, 2009). In addition, it was found that the mRNA expression of Bax in the rumen was inconsistent with that the protein expression of Bax. This phenomenon was more common in eukaryotic gene expression, which was caused by many factors in the process of mRNA translation. Therefore, combined with the expression of goat growth index, metabolic index, and apoptosis‐related genes, it was suggested that the ratio of ramie to alfalfa should not exceed 35% in goat feeding.

Caspase‐3 is a protease that can activate endonuclease, specific cleavage sequence of nucleosome connection, DNA breaks into fragments of 180–200 bp size. Meanwhile, destruction of cytoskeletal proteins, extracellular matrix proteins, nuclear proteins and so on, the cells lost their normal morphology and eventually induced the cells to apoptosis (Wang et al., 2015). In many types of cells, Caspase‐3 is essential for the completion of apoptosis and the formation of apoptotic bodies that produce nuclear and morphological changes (Bojes et al., 1999). The Caspase‐3 activity was not affected by adding different levels of ramie in the rumen and jejunum in the current study. One possible explanation was that apoptosis was not all dependent on the implementation of Caspase‐3 (Liu et al., 2016). Therefore, we speculated that ramie might induce apoptosis in GIT of goats in a caspase‐independent manner and the speculation was urgently needed for further research.

## 5 CONCLUSION

In conclusion, ramie with high substitution rate might induce the apoptosis in GIT of goats in a caspase‐independent manner, and ramie should not replace alfalfa more than 35% in the process of goat feeding. Further studies are warranted to examine the exact molecular mechanism of apoptosis induced by ramie in goats.

## CONFLICT OF INTEREST

The authors declare that there is no conflict of interest associated with the paper.

## ETHICAL APPROVAL

The experiments were conducted according to the animal care guidelines of the Animal Care Committee, Institute of Subtropical Agriculture, Chinese Academy of Sciences.
